# The Effect of Uridine on the State of Skeletal Muscles and the Functioning of Mitochondria in Duchenne Dystrophy

**DOI:** 10.3390/ijms231810660

**Published:** 2022-09-13

**Authors:** Mikhail V. Dubinin, Vlada S. Starinets, Natalia V. Belosludtseva, Irina B. Mikheeva, Yuliya A. Chelyadnikova, Daria K. Penkina, Alexander A. Vedernikov, Konstantin N. Belosludtsev

**Affiliations:** 1 Department of Biochemistry, Cell Biology and Microbiology, Mari State University, pl. Lenina 1, 424001 Yoshkar-Ola, Russia; 2 Laboratory of Mitochondrial Transport, Institute of Theoretical and Experimental Biophysics, Russian Academy of Sciences, Institutskaya 3, 142290 Pushchino, Russia

**Keywords:** Duchenne muscular dystrophy, skeletal muscle, mitochondria, uridine, mitochondrial dysfunction, lipid peroxidation, potassium transport

## Abstract

Duchenne muscular dystrophy is caused by the loss of functional dystrophin that secondarily causes systemic metabolic impairment in skeletal muscles and cardiomyocytes. The nutraceutical approach is considered as a possible complementary therapy for this pathology. In this work, we have studied the effect of pyrimidine nucleoside uridine (30 mg/kg/day for 28 days, i.p.), which plays an important role in cellular metabolism, on the development of DMD in the skeletal muscles of dystrophin deficient *mdx* mice, as well as its effect on the mitochondrial dysfunction that accompanies this pathology. We found that chronic uridine administration reduced fibrosis in the skeletal muscles of *mdx* mice, but it had no effect on the intensity of degeneration/regeneration cycles and inflammation, pseudohypetrophy, and muscle strength of the animals. Analysis of TEM micrographs showed that uridine also had no effect on the impaired mitochondrial ultrastructure of *mdx* mouse skeletal muscle. The administration of uridine was found to lead to an increase in the expression of the *Drp1* and *Parkin* genes, which may indicate an increase in the intensity of organelle fission and the normalization of mitophagy. Uridine had little effect on OXPHOS dysfunction in *mdx* mouse mitochondria, and moreover, it was suppressed in the mitochondria of wild type animals. At the same time, uridine restored the transport of potassium ions and reduced the production of reactive oxygen species; however, this had no effect on the impaired calcium retention capacity of *mdx* mouse mitochondria. The obtained results demonstrate that the used dose of uridine only partially prevents mitochondrial dysfunction in skeletal muscles during Duchenne dystrophy, though it mitigates the development of destructive processes in skeletal muscles.

## 1. Introduction

Duchenne muscular dystrophy is one of the most common forms of hereditary neuromuscular pathologies with an incidence of about one in 3500–5000 newborns, primarily boys [1]. This disease is accompanied by the degeneration of muscle fibers, inflammation, the replacement of functional tissue with connective tissue (fibrosis), and as a result, a decrease in the patient’s mobility. Subsequently, this leads to the progressive wasting of the locomotor and respiratory muscles, with consequent chronic ventilatory failure, as well as cardiomyopathy, which are the main causes of death [2].

One of the important secondary signs of DMD is considered to be systemic metabolic impairment, which leads to a decrease in the production of ATP; this is necessary for the operation of the contractile apparatus and the normal functioning of the muscle fiber [3,4]. In particular, the dystrophin deficient skeletal muscle of DMD patients and animal models show the reduced efficiency of glycolysis enzymes [5,6], as well as the enzymes involved in the TCA cycle [6,7] and the electron transport chain (ETC) [8,9,10]. These two events are directly related to mitochondrial dysfunction. Indeed, these organelles show a significant decrease in the rate of mitochondrial oxidative phosphorylation, which is also accompanied by a decrease in the efficiency of transporting and accumulating Ca^2+^, ROS overproduction, and the depolarization of organelles; this leads to mitochondrial permeability transition pore (PTP) opening and subsequent muscle fiber death, as well as the development of severe necrotic inflammation [9,11,12]. Given the systemic nature of DMD, the presence of many potential therapeutic targets, and the current imperfection of genetic approaches that can improve dystrophin expression, it is not surprising that there are many secondary therapeutic options for this pathology [13]. In particular, supplementary nutriceuticals that target and augment the bioenergetic expansion of the cellular metabolic pathways involved in energy generation have been widely investigated for their therapeutic efficacy in DMD treatment [4].

Our attention was drawn to pyrimidine nucleoside uridine, which plays an important role in many processes in mammalian organisms [14]. It is used as a building block for RNA biosynthesis, glycogen deposition, and it is involved in xenobiotic detoxification and cellular signaling processes. Uridine metabolism is associated with glucose homeostasis and lipid and amino acid exchange [14,15,16,17]. The effect of uridine on the functioning of the mitochondrial ETC has been also demonstrated [17]; therefore, a change in the concentration of uridine in the blood and the tissues of the human and animal body can lead to drastic changes in systemic energy homeostasis.

There are fragmentary data on the possibility of using uridine when treating DMD. In particular, the use of a cocktail of nucleosides and nucleotides containing adenosine, AMP, ADP, ATP, guanosine, guanosine monophosphate, uridine, uridine monophosphate, and cytidine monophosphate in the treatment of boys with DMD has been reported, and some improvement in enzymatic and functional capacity was shown [18]. The positive effects of this nucleoside on myocardial injuries in rat models with acute ischemia and ischemia/reperfusion is also known. It is assumed that its effects may be due to the mitigation of oxidative stress through the activation of the mitochondrial ATP-dependent K^+^ channel [19,20,21,22].

In this work, we have studied the effect of the intraperitoneal administration of uridine (30 mg/kg/day for 28 days) on the development of destructive processes in the skeletal muscles of model dystrophin deficient *mdx* mice. Taking into account the known role of uridine in the regulation of energy homeostasis, we also evaluated the effect of this nutraceutical on the mitochondrial dysfunction that accompanies this pathology, as well as the violation of the ultrastructure of organelles and the malfunctions that occur within the mitochondrial quality control system.

## 2. Results

### 2.1. Effect of Uridine Treatment on the Somatic and Biochemical Characteristics of Mice and Skeletal Muscle Health

**Figure 1:**
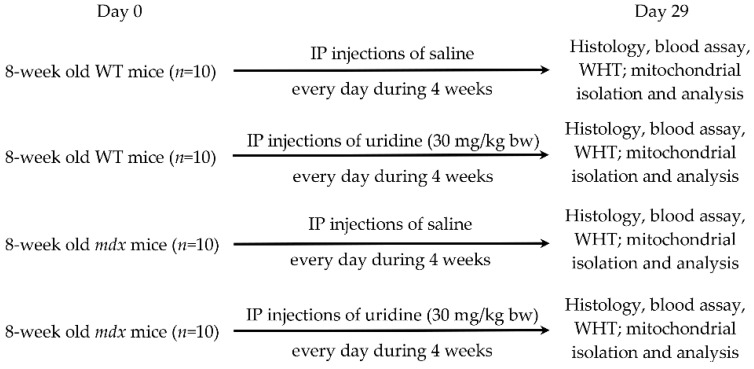
The experimental protocol of the study. IP: intraperitoneal; WHT: wire hanging test.

In the present work, we administered uridine at a dose of 30 mg/kg to wild type and *mdx* mice every day for 28 days (Figure 1). This dose and regimen were chosen because high chronic doses of uridine are known to induce the development of insulin resistance and obesity, which negatively affects animal health [23]. In this case, it should be noted that obesity also accelerates the progression of DMD, so calorie restriction is required [24]. Finally, this dose of uridine has been shown to work well on myocardial pathology for ischemia/reperfusion [22].

**Figure 2:**
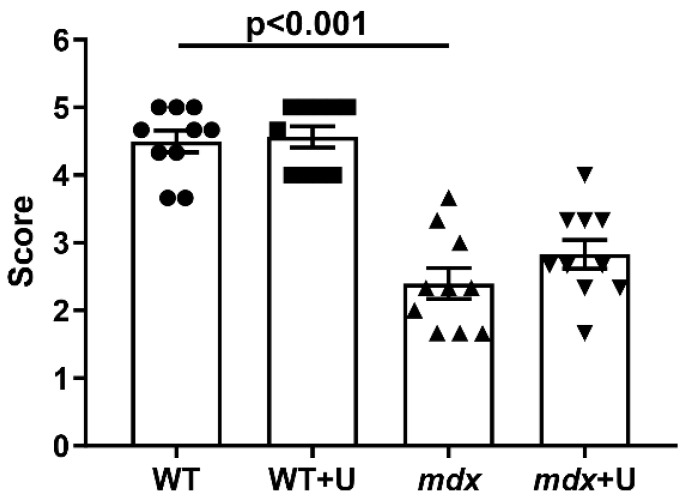
The results of a wire hanging test, reflecting the effect of uridine administration on muscle function and endurance in the studied groups of mice. The data are presented as means ± SEM (*n* = 10).

**Figure 3:**
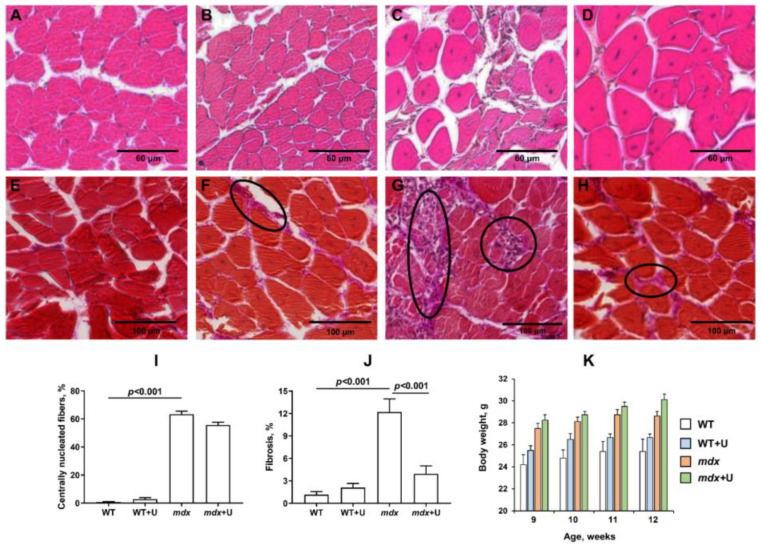
Representative histology images of skeletal muscle tissue (quadriceps) showing myofibers containing the centrally located nuclei (**A**–**D**) and fibrotic area (**E**–**H**, outlined in black ovals) in the WT (**A**,**E**), WT+U (**B**,**F**), *mdx* (**C**,**G**), and *mdx*+U (**D**,**H**) groups of mice. The bars are equal to 60 μm (**A**–**D**) and 100 μm (**E**–**H**). Diagrams (**I**) and (**J**) show the percentage of myofibers containing centrally located nuclei and the amount of interstitial fibrosis in the skeletal muscles of animals. The data are presented as means ± SEM (*n* = 5). The level of fibrosis was evaluated as the percentage ratio of the haematoxylin van Gieson (HvG) staining areas (pink) that represented collagen and other connective tissue elements in the common area of the tissue, wherein at least 10 sections were analyzed for each sample. Quantitative data of centrally nucleated fibers that underwent H&E staining are from, on average, 8–10 unique visual fields from each animal, and they are expressed as a percentage of the total number of myofibers counted. Panel (**K**) shows body weight gain in experimental groups of mice. The data are presented as means ± SEM (*n* = 10).

Dystrophin deficient mice have the following distinctive features that reflect the development of the pathology: decreased muscle strength and endurance, high levels of enzymes (which are markers of inflammation), as well as the intensive development of muscle degeneration and fibrosis, accompanied, among other things, by pseudohypertrophy, leading to accelerated weight gain. All these phenomena are also observed in the present work. Indeed, *mdx* mice show a decreased ability to hold onto a horizontal bar in the wire hanging test (Figure 2). In addition, the level of diagnostic enzymes present in the blood serum during muscle inflammation (creatine kinase, AST, and LDH) is significantly increased (Table 1). This is also accompanied by a significant increase in the level of centrally nucleated fibers (Figure 3A–D,I), a hallmark feature of ongoing cycles of degeneration and regeneration in muscles, as well as an increase in the level of fibrosis (Figure 3E–H,J), thus indicating the replacement of functional muscle tissue with connective tissue elements. Due to the high activity that occurs during these processes, *mdx* mice exhibit pseudohypertrophy, thus leading to accelerated weight gain (Figure 3K).

The administration of uridine does not significantly affect most of these parameters in both *mdx* mice and wild type animals. The muscle strength of the animals, the level of inflammatory markers, and the intensity of degeneration/regeneration cycles in mice do not change. We only noted a significant decrease in the level of fibrosis in the skeletal muscles of uridine treated *mdx* mice. In addition, the administration of uridine not only fails to suppress the development of pseudohypetrophy, but rather, it leads to increased weight gain in mice, which is especially noticeable in the case of wild type animals.

### 2.2. Effect of Uridine on the Ultrastructure of Skeletal Muscle Mitochondria

**Figure 4:**
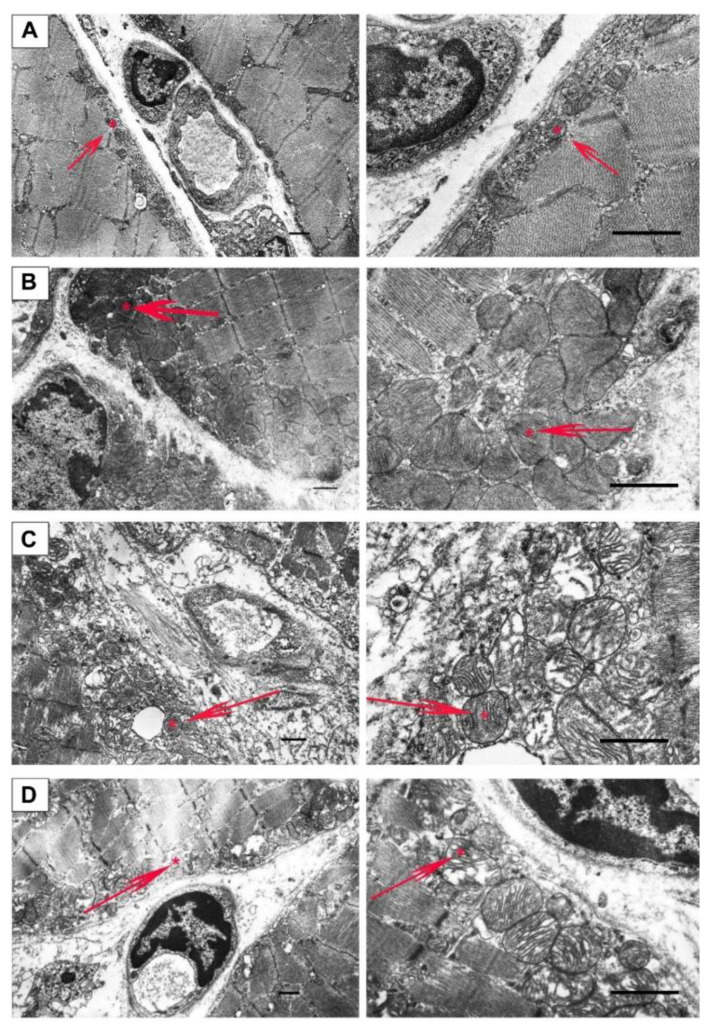
Representative transmission electron micrographs of mouse skeletal muscle (quadriceps) sections from WT (**A**), WT+U (**B**), *mdx* (**C**), and *mdx*+U (**D**) groups showing the quality of the ultrastructure of the subsarcolemmal population of mitochondria (highlighted by red arrows). An asterisk marks the same mitochondria at low and high magnification. The bars are equal to 1 μm.

**Figure 5:**
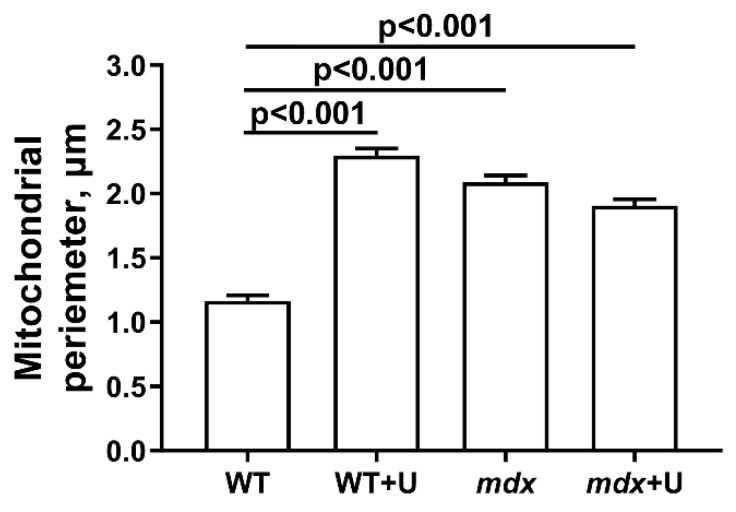
Perimeter of the mitochondria (μm) of the skeletal muscles of experimental animals. The number of examined fields of view was 50–70 in each group. The data are presented as means ± SEM.

Figure 4 and Figure 5 show electron microscopy data and the mean perimeters of the subsarcolemmal population of skeletal muscle mitochondria in mice (Figure 4, red arrows). In the control preparations, mitochondria are characterized by a low distribution density under the sarcolemma. They have a dense matrix with prominent, well-packed cristae (Figure 4A). In the WT+U group, we marked areas of mitochondrial proliferation. Unusually large mitochondria appear, but their matrix density and cristae packing do not differ from wild type mice (Figure 4B). Preparations of *mdx* mice show a sharp increase in the number of mitochondria under the sarcolemma with signs of severe destruction (Figure 4C). In particular, we observed hypertrophy of mitochondria due to their swelling (Figure 5) and a disturbance of the cristae structure and matrix density, which became clearer and vacuolated. Accumulations of glycogen and lysosomes are often observed around pathologically altered mitochondria. In the *mdx*+U group, the structure of the subsarcolemmal zone does not differ from the *mdx* group (Figure 4D). There are also massive accumulations of mitochondria under the sarcolemma with multiple structural disturbances. Thus, uridine does not affect the disturbed mitochondrial ultrastructure in the skeletal muscles of *mdx* mice, and moreover, it causes a change in the size of the mitochondria in the muscles of wild type animals.

### 2.3. Effect of Uridine on the mRNA Expression of Proteins Responsible for Mitochondrial Dynamics, Biogenesis, and Mitophagy

**Figure 6:**
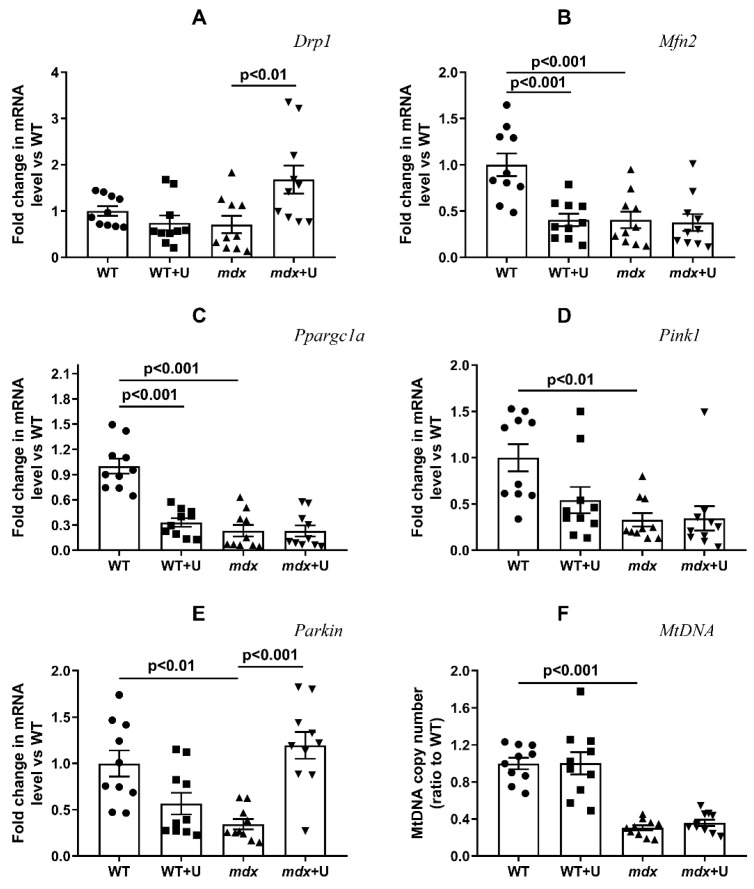
Effect of uridine on mitochondrial dynamics, biogenesis and mitophagy in skeletal muscle. The relative mRNA levels of *Drp1* (**A**), *Mfn2* (**B**), *Ppargc1a* (**C**), *Pink1* (**D**), *Parkin* (**E**), and mtDNA (**F**) in the skeletal muscles of experimental animals. The data are presented as means ± SEM (*n* = 10).

It is known that Duchenne dystrophy is accompanied by rearrangements in the systems responsible for mitochondrial fusion/fission, mitochondrial biogenesis, and mitophagy [25,26,27]. One can see that in the present work, the skeletal muscles of *mdx* mice show a decrease in the expression of the *Mfn2*, *Ppargc1a*, *Pink1*, and *Parkin* genes (Figure 6). This may indirectly indicate a decrease in the intensity of the processes concerned with mitochondrial fission and organelle biogenesis, as well as mitophagy, which are controlled by proteins that are encoded by the corresponding genes mentioned above. The expression of the *Drp1* gene does not change in Duchenne dystrophy. Treatment with uridine leads to a significant increase in *Drp1* gene expression in the mitochondria of *mdx* mice, whereas the level of *Mfn2* gene expression does not change, which may indirectly indicate an increase in the intensity of organelle fission. At the same time, we noted a decrease in *Mfn2* expression in the skeletal muscles of wild type mice treated with uridine. Uridine does not affect the expression of the *Ppargc1a* gene in *mdx* mice, which encodes a protein responsible for organelle biogenesis, though it significantly reduces the expression of this gene in the skeletal muscles of wild type mice, which may also indicate a decrease in organelle biogenesis. An estimation of the level of expression of the genes responsible for mitophagy shows that the administration of uridine does not affect the expression of the *Pink1* gene; instead, it leads to the normalization of *Parkin* expression in the skeletal muscles of *mdx* mice. Thus, uridine can activate mitophagy in the muscles of dystrophin deficient animals.

It is known that, along with the suppression of mitochondrial biogenesis, the skeletal muscles of dystrophin deficient mice are characterized by a decrease in the total level of mtDNA [26]. This was also observed in our study, and the administration of uridine does not affect this parameter (Figure 6F).

### 2.4. The Effect of Uridine on the Functioning of Skeletal Muscle Mitochondria in Dystrophin Deficient Mice

Violations of the ultrastructure of skeletal muscle mitochondria and the quality control system in Duchenne dystrophy is also accompanied by mitochondrial dysfunction. This is manifested in a decrease in the efficiency of oxidative phosphorylation, which is necessary for the production of ATP. Indeed, Table 2 shows the decrease in respiration rate in the ADP-stimulated state (State 3), as well as the maximal respiration rate in the presence of the protonophorone uncoupler 2,4-dinitrophenol (DNP; State 3U_DNP_) in *mdx* mouse skeletal muscle mitochondria compared with wild type animals. Treatment with uridine leads to a significant decrease in the state 4 respiration rate of *mdx* mice mitochondria, and there is also a tendency for the rate of oxygen consumption to further decrease in states 3 and 3U_DNP_. Moreover, in the case of wild type animals, the administration of uridine leads to a significant decrease in the rate of mitochondrial respiration in states 3 and 3U_DNP_; thus, uridine suppresses respiration and oxidative phosphorylation in skeletal muscle mitochondria, which is most pronounced in wild type animals, who exhibit higher respiratory rates when compared with *mdx* mice.

**Figure 7:**
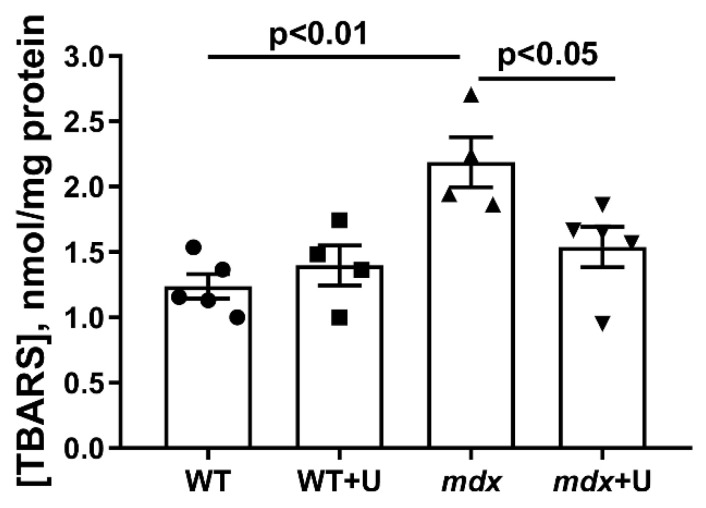
The effect of uridine on lipid peroxidation in mitochondria. Lipid peroxidation was assessed by the level of TBARS (mainly represented by MDA) in the skeletal muscle mitochondria of experimental groups of animals. The data are presented as means ± SEM (*n* = 4–5).

One of the triggers of skeletal muscle destruction in DMD is oxidative stress, which is manifested, among other things, by the development of lipid peroxidation in mitochondria [9,26]. Indeed, the mitochondria of *mdx* mice show an increased level of malondialdehyde (MDA) (Figure 7). In this case, the administration of uridine leads to the normalization of the MDA level.

**Figure 8:**
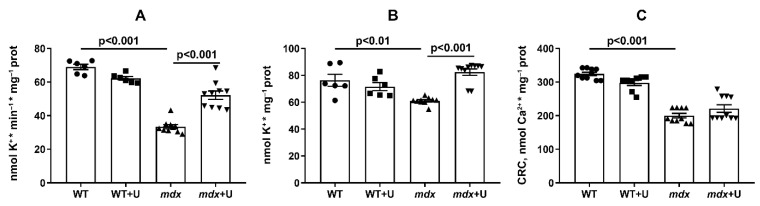
Assessment of the transport and level of potassium and calcium ions in the skeletal muscle mitochondria of mice in the experimental groups. The rate of the DNP-induced release of K^+^ from the skeletal muscle mitochondria of experimental animals (**A**). The total content of K^+^ in the skeletal muscle mitochondria of experimental animals (**B**). Ca^2+^ retention capacity of the skeletal muscle mitochondria of the experimental animals (**C**). The data are presented as means ± SEM (*n* = 6–10).

Uridine is known to be a precursor of uridine 5’-diphosphate (UDP), an activator of the mitochondrial ATP-dependent potassium channel (mitoK_ATP_) [28]; therefore, in the following section, we estimated the rate of the DNP-induced release of K^+^ from skeletal muscle mitochondria, which was mediated by the inversion of the mitoK_ATP_ [29]. One can see that DMD exhibits a significant (twofold) decrease in the rate of K^+^ release from mitochondria (Figure 8A). Moreover, we observed a decrease in the total level of K^+^ in the skeletal muscle mitochondria of *mdx* mice (Figure 8B). The use of uridine leads to a significant increase in both the rate of the DNP-induced release of K^+^ from the mitochondria of *mdx* mice and the total level of this ion in mitochondria.

The development of DMD is also associated with a decrease in calcium retention capacity and mitochondrial resistance to PTP opening [11,12,26]. Indeed, skeletal muscle mitochondria from *mdx* mice show an approximate 1.6 fold reduction in Ca^2+^ retention capacity (Figure 8C). Treatment with uridine has no effect on this parameter.

## 3. Discussion

Dysregulation of energy metabolism is considered one of the hallmarks of Duchenne muscular dystrophy. The administration of supplementary nutraceuticals in some cases may reduce the severity of the disease and improve the quality of life of patients [4]. One of these nutraceuticals is the nucleoside uridine, which plays an important role in the regulation of cellular function and energy metabolism, and in particular, in carbohydrate, protein, and lipid metabolism, and the biosynthesis of nucleic acids [14,15,16,17]. Moreover, uridine may affect the functioning of mitochondria [17]. In the present work, we evaluated the effect of the prolonged administration of uridine (30 mg/kg for 28 days) on the development of Duchenne muscular dystrophy in an *mdx* mouse model.

Previously, it has been shown that intravenous administration of uridine, in combination with a number of other nucleotides and precursors, improves the enzymatic and functional capacity of DMD patients [18]. Here, we found that uridine only partially alleviates muscle pathology by reducing the level of fibrosis in the skeletal muscles of *mdx* animals (Figure 3). DMD fibrosis is known to show the excessive or unregulated deposition of extracellular matrix (ECM) components, and it is a particular hallmark of the pathology’s development [30]. In this regard, the reduction of fibrosis may indicate an improvement in the control of the deposition of ECM components. Fibrosis reduction is actively used as an adjunct therapy in DMD, as it maintains the amount of target muscle available for therapy and repair [30]; however, in our study, this was not accompanied by a change in the intensity of degeneration/regeneration cycles and inflammation in *mdx* mice, nor the muscle strength of the animals (Figure 2 and Figure 3, Table 1). The level of pseudohypetrophy also does not change (Figure 3K). Moreover, mice treated with uridine show a trend (*p* > 0.05) towards enhanced body weight gain. We hypothesized that the latter is due to the known ability of uridine to increase muscle and liver glycogen synthesis, as well as to inhibit lipolysis in adipose tissue [31,32].

It is known that some of the positive effects of uridine in ischemic heart injury, as well as other pathologies, may result from the improved functioning of the mitochondrial network [19,20,21,22]. Based on this, the main objective of our study was to determine the effect of long-term administration of uridine on mitochondrial dysfunction in dystrophin deficient animals. It can be seen that uridine has no significant effect on the disturbed ultrastructure of the skeletal muscles and mitochondria in *mdx* mice (Figure 4 and Figure 5). At the same time, we noted the effect of uridine administration on the functioning of systems responsible for mitochondrial biogenesis, such as mitochondrial dynamics and mitophagy in animal skeletal muscles, which was reflected in the expression of the corresponding genes (Figure 6). Uridine has no effect on the reduced expression of the *Ppargc1a* gene in *mdx* mice, which is responsible for the synthesis of the PGC1α protein, the main regulator of mitochondrial biogenesis. Moreover, we noted a decrease in the expression of the *Ppargc1a* gene in uridine treated wild type mice, which may indirectly indicate the suppression of organelle biogenesis. On the other hand, uridine increases the expression of the *Drp1* gene in the skeletal muscles of *mdx* mice, encoding a protein responsible for mitochondrial fission. In contrast, the expression level of the *Mfn2* gene, which encodes a protein involved in organelle fusion and is downregulated in the muscles of *mdx* mice, does not change. Moreover, uridine reduces *Mfn2* expression in wild type mice. All of these events may favor organelle fission in the skeletal muscles of uridine-treated mice. Interestingly, uridine promotes an increase in the expression of the *Parkin* gene, which is involved in the regulation of mitophagy in the skeletal muscles of *mdx* mice. This may indirectly indicate the stimulation of mitophagy in uridine-treated *mdx* animals, which may contribute to the elimination of defective mitochondria; however, in the absence of an effect on organelle biogenesis, this does not lead to a change in the level of mtDNA in the skeletal muscles of dystrophin deficient animals, which was significantly reduced compared to the control.

Violation of the structure and dynamics of organelles during the development of DMD is also accompanied by the suppression of the OXPHOS system of skeletal muscle mitochondria and oxidative stress, as evidenced by the data on oxygen consumption by organelles in different functional states (Table 2), as well as an increase in the content of malondialdehyde (Figure 7). Uridine causes a significant decrease in the state 4 respiration rate of mitochondria in *mdx* mice, thus contributing to the restoration of the respiratory control ratio. We noted a tendency towards a decrease in the rate of respiration in the state 3 and 3U_DNP_ in uridine-treated *mdx* animals, and in the case of wild type animals, this effect is statistically significant. It is important that uridine significantly reduces the level of MDA in the skeletal muscle mitochondria of *mdx* mice, which may indicate a mitigation of oxidative stress.

The decrease in the MDA level under the influence of uridine may be due to the well-known mild uncoupling effect of this agent, which is based on the activation of potassium transport in mitochondria [22]. Indeed, it can be seen that the skeletal muscle mitochondria of *mdx* mice are characterized by a decrease in the rate of potassium transport and the total level of this ion (Figure 8A,B). It is believed that the transport of K^+^ through the inner mitochondrial membrane is mediated by potassium transporters, including the mitochondrial ATP-dependent K^+^ channel. Uridine is the precursor of UDP, the activator of this channel [28,33,34]. Moreover, it is notable that uridine-treated *mdx* animals demonstrate a significant increase in both the rate of potassium ion transport and the total level of this ion in skeletal muscle mitochondria. Here, it is important to note the previously identified disruption in the organization and functioning of the ATP-dependent K^+^ channel in *mdx* mice, at least in cardiomyocytes [35]. One could assume that uridine is able to partially restore the activity of this channel in mitochondria, which has a positive effect on potassium homeostasis in these organelles.

Another phenomenon closely associated with the development of mitochondrial dysfunction and destructive processes in DMD muscle tissue is the calcium dependent mitochondrial PTP [11,12,26]. It is known that the opening of the PTP, which represents a protein mega channel that is formed in the inner and outer mitochondrial membranes by cyclophilin D, adenylate translocator, and ATP synthase, reduces the ability of mitochondria to efficiently accumulate calcium ions and regulate ion homeostasis in skeletal muscle [36,37]. Improving the calcium retention capacity with the PTP inhibitor alisporivir has previously been shown to normalize the structural and functional characteristics of *mdx* mitochondria and mitigate the effects of the pathology [26,38]. The administration of uridine to *mdx* animals does not lead to a significant increase in the CRC parameter (Figure 8C), thus reflecting the resistance of mitochondria to the induction of the Ca^2+^ dependent PTP. In this regard, we can conclude that under our experimental conditions, despite the improvement in the functioning of the mitochondrial K^+^ channel and the decrease in the level of MDA, uridine is not able to affect the opening of the PTP in DMD skeletal muscle mitochondria.

## 4. Materials and Methods

### 4.1. Experimental Animals

Male mice of the C57BL/10 (wild type, WT) and dystrophin deficient *mdx* (C57BL/10ScSn-mdx) were used in this work. All the animals were purchased from the Animal Breeding Facility, Branch of the Shemyakin and Ovchinnikov Institute of Bioorganic Chemistry, Russian Academy of Sciences, Russia (IBCh RAS Unique Research Device “Bio-model”, Pushchino, Russia). Upon arrival, mice were singly housed and given a minimum of 72 h to acclimatize before experiments were performed. All animals were provided access to standard chow and water ad libitum. Mitochondrial isolation was performed on fresh samples of skeletal muscle. The rest of the tissue was stored at −80 °C until analyzed.

### 4.2. Uridine Administration

*Mdx* and WT mice (eight weeks old) were injected interperitoneally with uridine (Sigma-Aldrich, St. Louis, MO, USA), and were resuspended in sterile saline every day for up to four weeks (30 mg/kg body weight). Sham-injected controls received saline alone. The mice were then killed and tissues were removed for analysis.

### 4.3. Blood Analysis

At the end of the treatment period, all mice were sacrificed. Blood was collected to analyze the activity of creatine kinase, AST, and LDH using the appropriate reagent kits (Vector-Best, Novosibirsk, Russia) and Multiskan GO plate reader (Thermo Fisher Scientific, Waltham, MA, USA).

### 4.4. Histological Examination of Skeletal Muscle Tissues

To examine the degree of severity of the histological changes, samples of skeletal muscle tissue (quadriceps, five samples in each experimental group) were fixed in a neutral buffered 10% formalin, and the specimens were impregnated in paraffin wax. The paraffin blocks were cut using a microtome MC-2, into serial sections, at a 5 μm thickness. The slides obtained from each specimen were stained with the haematoxylin and eosin (H&E) and haematoxylin van Gieson (HvG) stain protocol. Examinations of the slides were carried out using an EVOS M5000 imaging system (Thermo Fisher Scientific, Waltham, MA, USA). All histological images were analyzed using free ImageJ software. The level of fibrosis in the muscles of mice was evaluated as the percentage ratio of the HvG staining areas (pink) which represented collagen and other connective tissue elements in the common area of the tissue on the histological slides, wherein at least 10 sections were analyzed for each organ sample. Quantitative data concerning the centrally nucleated fibers (CNF) from H&E staining are from, on average, 8–10 unique visual fields (20× objective) from each animal, and they were expressed as a percentage of the total number of myofibers counted.

### 4.5. Wire-Hanging Test

Muscle function and the endurance of the mice were assessed using a wire-hanging test. For this purpose, each animal was placed on a 3 mm string (38 cm long and 49 cm above a soft surface to cushion animals that fall off). The mouse was held on the string by its front paws and hung for 30 s. The scoring of the test results was carried out according to a well-known approach [39]: hanging for 1–5 s = 1 point, hanging for 6–10 s = 2 points, hanging for 11–20 s = 3 points, hanging for 21–30 s = 4 points, and hanging for 30 s = 5 points. Placing one forepaw on a bar support without falling = 5 points. An animal that repeatedly failed before the 5 s elapsed received only 1 point. Each animal was allowed three attempts, and the average was used for the final calculation.

### 4.6. Transmission Electron Microscopy

Samples of the skeletal muscle (quadriceps, two samples in each group) were fixed for 2 h in a 2.5% glutaraldehyde solution in 0.1 M PBS (pH = 7.4). The preparations were examined and photographed using a JEM-100B electron microscope (JEOL, Tokyo, Japan) and analyzed using Image Tool 3.0 software.

### 4.7. RNA Extraction, Reverse Transcription, and Quantitative Real-Time PCR

Total RNA was isolated from 100 mg of deep-frozen samples from the quadriceps of the mice using an ExtractRNA kit and its corresponding protocol (#BC032, Eurogen, Moscow, Russia). The real-time PCR was performed on a QuantStudio 1 (Thermo Fisher Scientific, Waltham, MA, USA) using the qPCRmix-HS SYBR reaction mixture (Eurogen, Moscow, Russia). The selection and analysis of gene-specific primers were performed using Primer-BLAST [40] (the oligonucleotide sequences are presented in Table 3). The relative level of expression of each gene was normalized to the level of *Rplp2* mRNA, and a comparative C_T_ method was used to quantify the results [41].

### 4.8. Quantification of Mitochondrial DNA

Total DNA (nuclear and mtDNA) was extracted from 10 mg of quadriceps using DNA-Extran 2 kit and protocol (Sintol, Moscow, Russia). Of the total DNA, 1 ng was taken for the reaction. The evaluation of mtDNA content in the samples was performed by PCR, as described in [42], and expressed as a mtDNA/nuclear DNA ratio. We selected the *ND4* gene of the mouse mitochondrial genome and the nuclear encoded *GAPDH* gene. A comparison of *ND4* DNA expression, relative to *GAPDH* DNA expression, gives a measure of the mtDNA copy number to nDNA copy number ratio. Primers for mtDNA and nDNA are presented in Table 3. The real-time PCR was performed with a QuantStudio 1 amplifier (Thermo Fisher Scientific, Waltham, MA, USA) using the qPCRmix-HS SYBR reaction mixture (Eurogen, Russia), which contained a commonly used fluorescent DNA binding dye SYBR Green II.

### 4.9. Skeletal Muscle Mitochondria Isolation and Determination of Functional Parameters

Mitochondria were isolated from skeletal muscle (quadriceps of both hindlimbs) tissue using the differential centrifugation method [11]. Final mitochondrial samples contained 25–35 mg of protein/mL, as determined by the Bradford method. The rate of O_2_ consumption by mitochondrial samples was measured using Oxygraph-2k (Oroboros instruments, Austria) [43]. The respiration medium contained 120 mM KCl, 5 mM NaH_2_PO_4_, 2.5 mM potassium malate, 2.5 mM potassium glutamate, and 10 mM Hepes/KOH (pH 7.4). Lipid peroxidation in mitochondria was assessed using the quantification of thiobarbituric acid-reactive substances (TBARS), which was represented mainly by malondialdehyde and some other minor aldehyde species [44]. Transport of K^+^ in mitochondria was determined by the rate of the DNP-induced release of K^+^ from the organelles using K^+^- selective electrode [29]. The medium contained 180 mM sucrose, 70 mM mannitol, 5 mM NaH_2_PO_4_, 1 μg/mL oligomycin, and 10 mM Tris/HCl, pH 7.4. Ca^2+^ transport and retention by mitochondria was recorded using arsenazo III color indicator at 675–685 nm and a plate reader Tecan Spark 10 M (Tecan, Switzerland) [45]. The medium contained 210 mM mannitol, 70 mM sucrose, 1 mM KH_2_PO_4_, 2.5 mM malate, 2.5 mM glutamate, 10 μM EGTA, 50 μM arsenazo III, and 10 mM HEPES-KOH (pH 7.4.). The total amount of the added Ca^2+^ ions that induced their spontaneous release from mitochondria due to the induction of the PTP opening was reflected in the calcium retention capacity (CRC) of the organelles.

### 4.10. Statistical Analysis

The data were analyzed using the GraphPad Prism 8.0.1 software (GraphPad Software Inc., San Diego, CA, USA) and were presented as the mean ± SEM of 4–10 biological replicates (excluding electron microscopy data). The results of the transmission electron microscopy analysis were presented as representative images from two biological replicates. The statistical significance of the differences between the experimental groups was evaluated using one-way analysis of variance (ANOVA) followed by the Tukey multiple comparison post hoc test.

## 5. Conclusions

The present work shows the effect of prolonged administration of nucleoside uridine (30 mg/kg for 4 weeks) on the progression of DMD and its concomitant mitochondrial dysfunction in skeletal muscle mitochondria. The following results were obtained: (1) uridine reduces the level of fibrosis in the skeletal muscles of *mdx* mice, but does not affect the intensity of degeneration/regeneration cycles and inflammation, nor the pseudohypetrophy and muscle strength of animals; (2) uridine at the dose used has no significant effect on the disturbed ultrastructure of the skeletal muscles and mitochondria of *mdx* mice; (3) uridine possibly favors the intensive fission of organelles in the skeletal muscles of *mdx* mice, thus increasing the expression of the *Drp1* gene, and causing an increase in the expression of the *Parkin* gene responsible for mitophagy, however, the level of mtDNA and the expression of the *Ppargc1a* gene responsible for organelle biogenesis do not change; (4) uridine has a weak effect on the dysfunctionality of the *mdx* mitochondrial OXPHOS system, and moreover, it suppresses it in the mitochondria of wild type mice. At the same time, uridine restores the functioning of potassium ion transport systems in *mdx* mice mitochondria and reduces the production of reactive oxygen species; however, this does not contribute to the normalization of the calcium retention capacity of organelles, and thus, the prolonged administration of uridine only partially restores the functioning of skeletal muscle mitochondria in Duchenne dystrophy and mitigates the development of destructive processes in skeletal muscles, subsequently reducing the level of fibrosis. This is not enough to significantly improve the quality of life of dystrophin deficient animals that still display the hallmarks of DMD—intense degeneration, inflammation, and calcium dysregulation; however, this approach, and the reduction of fibrosis can be used as an adjunctive therapy to maintain a sufficient amount of target muscle that may be available for therapy and repair.

## Figures and Tables

**Table 1:** The level of creatine kinase, AST, and LDH in the serum of experimental groups of mice.

Animals	Creatine Kinase	AST	LDH
	U/L		
WT *(n = 10)*	339.5 ± 57.6	26.7 ± 3.0	325.9 ± 25.4
WT+U *(n = 10)*	449.3 ± 59.4	28.7 ± 5.1	372.7 ± 32.5
*mdx (n = 10)*	854.3 ± 69.2 **	97.2 ± 9.6 **	1027.6 ± 103.7 **
*mdx*+U *(n = 10)*	823.6 ± 101.8 *	120.7 ± 12.6 **	1116.9 ± 145.4 **

Data are presented as means ± SEM; *n*, the number of experimental animals. * *p* < 0.01 versus control group (WT); ** *p* < 0.001 versus control group (WT).

**Table 2:** Parameters of respiration and oxidative phosphorylation of mouse skeletal muscle mitochondria in the experimental groups.

Group	V Respiration, nmol O_2_ * min^−1^ * mg^−1^ Protein				RCR	*ADP/O*
	State 2	State 3	State 4	State 3U_DNP_		
WT	24.1 ± 1.9	201.2 ± 6.7	45.1 ± 3.6	221.3 ± 9.1	4.6 ± 0.3	1.2 ± 0.1
WT+U	25.9 ± 2.3	156.2 ± 7.4 *	38.3 ± 1.9	175.8 ± 6.3 *	4.2 ± 0.3	1.3 ± 0.1
*mdx*	20.2 ± 1.4	171.4 ± 5.8 *	56.3 ± 8.3	187.3 ± 7.1 *	3.7 ± 0.5	1.1 ± 0.1
*mdx*+U	27.3 ± 2.7	154.7 ± 5.5 *	34.7 ± 2.8 #	174.0 ± 6.6 *	4.5 ± 0.3	1.3 ± 0.1

Mitochondria respiration was fueled by 2.5 mM glutamate and 2.5 mM malate. State 3 respiration was initiated by 100 µM ADP. The results are presented as means ± SEM (*n* = 6). * *p* < 0.05 versus control group (WT); # *p* < 0.05 versus *mdx* group.

**Table 3:** List of gene-specific primers for RT-PCR analysis.

Gene	Forward (5’→3’)	Reverse (5’→3’)
*Pink1*	TTGCCCCACACCCTAACATC	GCAGGGTACAGGGGTAGTTCT
*Parkin*	AGCCAGAGGTCCAGCAGTTA	GAGGGTTGCTTGTTTGCAGG
*D* *rp1*	TTACAGCACACAGGAATTGT	TTGTCACGGGCAACCTTTTA
*Mfn2*	CACGCTGATGCAGACGGAGAA	ATCCCAGCGGTTGTTCAGG
*Ppargc1a*	CTGCCATTGTTAAGACCGAG	GTGTGAGGAGGGTCATCGTT
*Rplp2*	CGGCTCAACAAGGTCATCAGTGA	AGCAGAAACAGCCACAGCCCCAC
*Nd4*	ATTATTATTACCCGATGAGGGAACC	ATTAAGATGAGGGCAATTAGCAGT
*Gapdh*	GTGAGGGAGATGCYCAGTGT	CTGGCATTGCTCTCAATGAC

## Data Availability

The data presented in this study are available upon request from the corresponding author.
